# Effect of body massage on increase of low birth weight neonates growth parameters: A randomized clinical trial 

**Published:** 2013-07

**Authors:** Sedighah Akhavan Karbasi, Motahhareh Golestan, Razieh Fallah, Mohammad Golshan, Zinabossadat Dehghan

**Affiliations:** 1 *Department of Pediatrics, Growth Disorders of Children Research Center, Shahid Sadoughi University of Medical Sciences, Yazd, Iran.*; 2 *Ali-ebn-Abitaleb School of Medicine, Islamic Azad University, Yazd Branch, Yazd, Iran.*; 3 *Shahid Sadoughi University of Medical Sciences, Yazd, Iran.*

**Keywords:** *Low birth weight*, *Massage*, *Weight*, *Height*, *Head Circumference*

## Abstract

**Background:** Admission of low birth-weight (LBW) neonates in neonatal intensive care unit (NICU) causes their deprivation of tactile and sensory stimulation.

**Objective:** The purpose of this study was to evaluate efficacy of body massage on growth parameters (weight, height and head circumference) gain velocity of LBW in Yazd, Iran.

**Materials and Methods:** A randomized clinical trial study was conducted on LBW neonates whom were admitted to NICU of Shahid Sadoughi Hospital, Yazd, Iran from March to December 2011. Neonates were randomly assigned to two groups. In group one, 20 neonates were received massage three times in a day for consecutive 14 days by their mothers. In group two, intervention consisted of standard and routine care as control group. The primary endpoints were efficacy in increase of mean of weight, height and head circumference that were evaluated 14 days after intervention, at ages one and two months. Secondary outcome was clinical side effects.

**Results: **17 girls and 23 boys with mean gestational age of 34.4±1.22 weeks were evaluated. In the body massage group, only weight at the age of two months was significantly higher than the control group (mean±SD: 3250±305 vs. 2948±121 gr, p=0.005). No adverse events were seen in the two groups.

**Conclusion:** Body massage might be used as an effective and safe non-medical intervention for increasing of weight gain velocity in LBW preterm neonates.

## Introduction

“Low birth weight (LBW or birth weight of less than 2500 gr) is one of the main determinants of neonatal and postnatal morbidity (1, 2). According to WHO statistics, the rate of LBW is 17% in the whole world, 6% in industrialized countries, 21% in developing countries and it is 10% in Islamic Republic of Iran (3). Based on the result of one study, the LBW rate in Yazd, Iran, is 8.4% (4). LBW is caused by preterm birth, intrauterine growth retardation or both "(1). 

LBW neonates may be admitted in neonatal intensive care unit (NICU) for birth weight less than 1500 gr or medical problems and NICU is noisy environment with policy of minimal touch to avoid acquired infection and it causes that premature neonates are deprived of tactile and sensory stimulation that is important in their growth outcome (2). Body massage as a non-medical intervention might have positive effect on physical and developmental growth of preterm and LBW infants including weight gain, decreased stress behavior, promotion of neurologic and neuromotor development, better infant-parent emotional bonding, improved sleep, reduced rates of nosocomial infection and thereby, decreased mortality of hospitalized infants (5-7).

Massage therapy has not any harmful effects and it can increase weight gain velocity of more than 30 weeks of gestation and medically stable neonates by different mechanisms. Weight gain is the most consistent parameter which is associated with massage therapy in neonates (5-9). Efficacy of massage on weight gain of LBW was evaluated in other Iranian studies (10-14). But this is the first study in Yazd, and the purpose of this study was to evaluate efficacy of body massage on growth parameters (weight, height and head circumference) gain velocity of LBW neonates.

## Materials and methods

A randomized single-blind clinical, open-label, parallel group study was conducted on LBW neonates whom were admitted to NICU of Shahid Sadoughi Hospital, Yazd, Iran from March to December 2011. Sample size was based on Z formula and a confidence interval of 95% with 80% power, type one error of 5%, with a standard deviation of 2.57 g/kg/day by massage in another study and an effect size (difference in weight gain velocity between the two groups) of 3 g/kg/day and allowing for a 5% loss to follow-up (15). 

Eligible participants included 40 newborns that had gestational age of 33-37 weeks, birth weight of 1500-1999 grams, who were without birth asphyxia and hypoxic ischemic insults, who were less than ten days, were medically stable and did not need any drug therapy and stayed in the hospital for at least 5 days after enrollment in the intervention. Exclusion criteria were multiple pregnancies, sepsis and meningitis, major congenital malformations, small for gestational age, chromosomal abnormalities, genetic syndromes, and serious complications such as intraventricular hemorrhage, severe respiratory distress, and necrotizing enterocolitis during NICU admission period. The trial used equal randomization and allocation ratio was 1:1 for the two groups (case and control). 

Simple randomization was done by a computer generated random numbers list which was prepared by an investigator with no clinical involvement in the trial. The intervention of massage was delivered by mothers and primary and secondary outcomes were assessed by the interne of research who was not informed of the intervention group assignment. Neonates mothers^,^ and physicians allocated to the intervention group were aware of the allocated arm. But, outcome assessors and data analysts were kept blind to the allocation.

Neonates were randomly assigned to two groups. In group one, 20 neonates massaged three times a day for 14 consecutive days. In group two, intervention consisted of standard and routine care only as control group. Each mother was trained for technique of massage, in the first day after delivery by a researcher. Compliance of mothers was checked regularly by the researchers during stay of newborns at hospital. 

"The body massage was done by mothers and each session massage was consisted ten minutes and for three times per day (in the morning, at noon and before bedtime) and it was done for two weeks. Massage was given in infant who was in a prone and supine position it was carried out from the neck and over both shoulders, upper back and then each of the two upper and lower limbs was separately massaged (except for face and head). Then massage was done in a supine position, chest, abdomen, upper limb, lower limb, palms and sole massaged separately ten gentle strokes was used in each area the massage" (16).

Growth parameters (weight, height and head circumference) of all neonates were measured 14 days after starting of body massage, at ages of one month and two months." All babies were weighted by infant digital weighing scale with sensitivity of 10 gram without diapers. The weighting scale was calibrated at regular intervals. The supine crown heel length was measured on the infantometer with the help of an assistant to the nearest millimeter in the recumbent position. The weighing scale and infantometer were Germany made Seca.

Head circumference was measured using flexible non-stretchable tape measure which runs from the supraorbital ridge to the occiput in the path as the maximum occipitofrontal circumference. To obviate errors due to interobsever variations, all measurement were made in Shahid Sadoughi Hospital and by the interne of research (1). The primary endpoints were efficacy in increase mean of weight, height and head circumference that evaluated at the end of intervention, at ages of one month and two months. Secondary outcome was clinical side effects in duration of follow up. 

Variables such age, sex, gestational age, route of delivery, age and educational level of mother were carefully recorded by medical records of mother and neonate. "Gestational age was calculated using the first day of the last normal menstrual period, estimated by obstetric sonography and the Dubowitz Scale" (4). Informed consent was taken from parents and the study has been approved by the Ethic Committee of Shahid Sadoughi University of Medical Sciences, Yazd, Iran. 

**Statistical analysis**

The data were analyzed using Statistical Package for the Social Sciences version 15 (SPSS, Chicago, IL, USA) statistical software. Chi-square test or Fisher exact test was used for data analysis of qualitative variables and mean values were compared using independent Student t-test. Differences were considered significant at p-values of less than 0.05.

## Results

**Figure 1:**
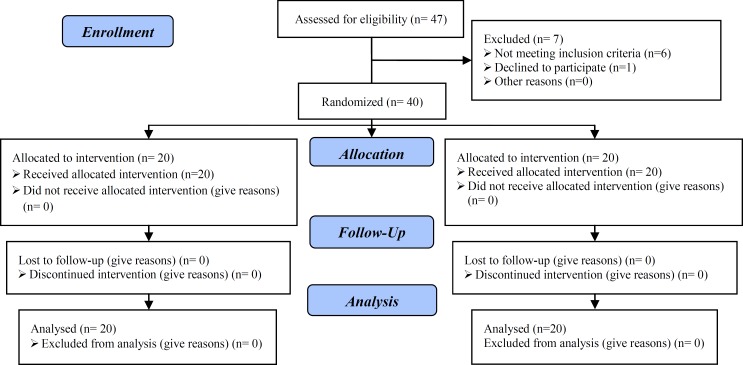
Consort flow diagram

Seven patients were excluded from the study and the design and conduct of this trial was straightforward, and we did not have any losses to follow-up (Figure 1). Finally, 40 neonates including 17 girls (42.5%) and 23 boys (57.5%) with mean gestational age of 34.4±1.22 weeks in two groups were evaluated. Comparison of some characteristics of the neonates is shown in table I which indicates that no statistically significant differences were seen from viewpoints of sex distribution, mean of gestational age, mean of birth weight, height and head circumference, mother educational level and mean of mother age in two groups.

Tables II, III and IV show comparison of mean of weight, height and head circumference of neonates at two weeks after intervention, the age of one month and two months that indicate in the body massage group, only weight at the age of two months was significantly higher than the control group (mean±SD: 3250±305 vs. 2948±121gr, p=0.005). No adverse events were seen in the two groups.

**Table I:** Comparison of some characteristics of neonates in groups

** Group**		**Massage**	**Only ** **standard ** **care**	**p-value**
**Data**				
Sex*				
	Girl	9	8	0.35
	Boy	11	12	
Maternal educational level*				
	Illiterate	1	0	0.43
	Primary - secondary school	8	7	
	High school	8	10	
	Higher education	3	3	
Gestational age in week (mean±SD)**		34.5±1.26	34.6±1.35	0.974
Birth weight in gram (mean±SD) **		1721±123	1539±513	0.406
Birth height in centimeter (mean±SD) **		41.62±1.39	41.83±1.41	0.969
Birth head circumference in centimeter (mean±SD) **		30.58±0.66	30.6±0.83	0.740
Mother age in year (mean±SD) **		24.3± 3.5	25.2 ± 3.7	0.321

* : Used statistical test: Chi-square test.

** : Used statistical test: Independent T-test

**Table II:** Comparison of mean of weight, height and head circumference of neonates at two weeks after intervention

** Group**	**Massage**	**Only ** **standard ** **care**	**p-value**
**Data**			
Weight in gram (mean ±SD)*	1879 ± 203	1700 ± 306	0.305
Height in centimeter (mean ±SD)	42.64 ± 1.38	42.78 ± 1.32	0.960
Head circumference in centimeter(mean ±SD)	31.06 ± 1.11	31.29 ± 0.49	0.214

* Used statistical test: student T-test

**Table III:** Comparison of mean of weight, height and head circumference of neonates at the age of one month

** Group**	**Massage**	**Only ** **standard ** **care**	**p-value**
**Data**			
Weight in gram (mean ±SD)*	2201 ± 93	2134 ± 354	0.05
Height in centimeter (mean ±SD)	44.01 ± 1.27	44.27 ± 1.33	0. 251
Head circumference in centimeter(mean ±SD)	32.16 ± 0.51	32.05 ± 0.97	0.204

* Used statistical test: student T-test

**Table IV:** Comparison of mean of weight, height and head circumference of neonates at the age of two months

** Group**	**Massage**	**Only ** **standard ** **care**	**p-value**
**Data**			
Weight in gram (mean ±SD)*	3250 ± 305	2948 ± 121	0.005
Height in centimeter (mean ±SD)	48.14 ± 1.25	47.88 ± 0.97	0.241
Head circumference in centimeter(mean ±SD)	33.28 ± 0.52	33.08 ± 1.07	0.141

* Used statistical test: student T-test

## Discussion

Massage of neonate that is defined as systematic application of touch, can be done by a trained person or mother. Researchers have reported that moderate pressure massage, especially with tactile kinesthetic stimulation, can improve weight gain velocity of LBW infants by different mechanisms such as increase in vagal activity, increased insulin release, reduced energy expenditure, increased gastric motility and better absorption of nutrients, decreased cortisol and norepinephrine serum level and less stress behavior of neonates (5-9). 

Massage therapy by both mothers and professionals have equivalent effects and in addition, mothers who massage their neonates have lower depression and anxiety symptoms (7). In present study, efficacy of body massage by mothers on increasing of mean of growth parameters (weight, height and head circumference) of LBW preterm neonates was evaluated and results showed that body massage was only effective on weight gain of LBW infants at the age of two months. 

Effectiveness of body massage in improvement of weight gain velocity of preterm or LBW neonates in different times have been reported in other studies (6-8, 10, 12, 13, 15-17). In Badiee *et al* study in Isfahan, Iran, medically stable 28-34 weeks neonates whom were massaged three times a day for five consecutive days by a trained nurse, had significantly more weight gain than those massaged by their mothers or control group (10). In Javadifar *et al* study, healthy 34-37 weeks neonates whom were massaged without or with coconut oil three times a day from the age of 3-17 days after birth by their mother, had significantly more weight gain than control group at the ages of 7 and 14 days (12).

In Diego *et al* study, preterm newborns whom received five days of massage therapy showed a 25% greater increase in weight gain than control group (8). In meta-analysis study of Vickers *et al*, body massage increased daily weight gain of neonates by 5.1 gr on average and also weight gain at 4-6 months (5). In a research in India, a trained person provided massages four times a day until the neonates were discharged and after discharge the massages were given by the mother until the infants were 31 days and results showed that in LBW preterm neonates, weight gain velocity in 31 days was significantly greater in the coconut oil massage as compared to massage with mineral oil and placebo (powder) groups (16). 

However, in Massaro *et al *study in Washington, only massage therapy with kinesthetic stimulation (passive limb movements) can improve weight gain of less than 1500 gr premature neonates and also, in Saeedi *et al* study in Neyshabur, Iran, only massage with coconut oil was effective in weight gaining of preterm newborns (18, 19). Possible explanations for these discrepancies are differences in: gestational age, method of massage, pressure of massage and person who administered massage. 

Efficacy of oral and tactile/kinesthetic stimulation in improvement of weight gain and motor function of preterm infants have been reported in Fucile *et al* study in Montreal (17). Some other studies concluded that only body massage with moderate pressure kinetic stimulation can significantly increase weight gain than light pressure (6, 20). In majority of other studies, height and head circumference of participants were not evaluated (6, 8, 10, 11, 13, 18). 

In present study body massage was not effective on height or head circumference growth which is in agreement to other studies (16, 21, 22). But, in Sankaranarayanan *et al* study, preterm LBW neonates whom were massaged with coconut oil, had a greater length gain velocity than placebo (powder) group and in Soriano *et al* study, preterm neonates whom were massaged with soybean oil, had a significant increase in anthropometric parameters at one month of age as compared to without massage group (16, 23). 

Therefore body massage with topical oils might be more effective in increase of height or head circumference growth velocity. 

## Conclusion

Based on the result of this study, body massage increased mean of weight of low birth weight preterm neonates at the age of two months and it can be used as a simple effective and safe non-medical intervention that can improve weight gain velocity of preterm low birth weight infants. 
